# Clinicopathological and prognostic significance of platelet-lymphocyte ratio (PLR) in gastric cancer: an updated meta-analysis

**DOI:** 10.1186/s12957-020-01952-2

**Published:** 2020-07-30

**Authors:** Xunlei Zhang, Wenjing Zhao, Yang Yu, Xue Qi, Li Song, Chenfei Zhang, Guoxing Li, Lei Yang

**Affiliations:** 1 Department of Oncology, Tumor Hospital Affiliated to Nantong University, Nantong, 226300 Jiangsu China; 2 Cancer Research Center Nantong, Tumor Hospital Affiliated to Nantong University, Nantong, 226300 Jiangsu China; 3 Department of General Surgery, Tumor Hospital Affiliated to Nantong University, Nantong, 226300 Jiangsu China; 4 Department of Oncology, Nantong Liangchun Hospital of Traditional Chinese Medicine, Nantong, 226300 Jiangsu China

**Keywords:** Platelet, Lymphocyte, PLR, Gastric cancer, Meta-analysis

## Abstract

**Background:**

Pre-treatment PLR (platelet-lymphocyte ratio) was reported to be associated with the prognosis in gastric cancer (GC), but the results remain inconclusive. This meta-analysis aimed to investigate the prognostic potential of the pre-treatment PLR in gastric cancer.

**Methods:**

We performed a systematic literature search in PubMed, Embase, and the Cochrane Library to identify eligible publications. The hazard ratio (HR)/odds ratio (OR) and its 95% confidence (CI) of survival outcomes and clinicopathological parameters were calculated.

**Results:**

A total of 49 studies (51 cohorts), collecting data from 28,929 GC patients, were included in the final analysis. The pooled results demonstrated that the elevated pre-treatment PLR was significantly associated with poor overall survival (OS) (HR 1.37, 95% CI 1.26–1.49, *p* < 0.001; *I*^2^ = 79.90%, *P*_h_ < 0.001) and disease-free survival (DFS) (HR 1.52, 95% CI 1.22–1.90, *p* < 0.001, *I*^2^ = 88.6%, *P*_h_ < 0.001). Furthermore, the patients with the elevated PLR had a higher risk of lymph node metastasis (OR = 1.17, 95% CI 1.02–1.33, *p* = 0.023), serosal invasion (T3+T4) (OR = 1.34, 95% CI 1.10–1.64, *p* = 0.003), and increased advanced stage (III+IV) (OR = 1.20, 95% CI 1.06–1.37, *p* = 0.004).

**Conclusions:**

An elevated pre-treatment PLR was a prognostic factor for poor OS and DFS and associated with poor clinicopathological parameters in GC patients.

## Background

Gastric cancer (GC) is a kind of common malignant tumor and one of the main causes of cancer-related mortality and morbidity worldwide [1]. Majority of the patients are diagnosed at an advanced stage due to no symptoms in the early stage. Complete or partial resection is the only potential curative treatment. However, the high recurrence and metastasis after resection lead to the poor level of 5-year survival rate [2]. For individual patients with different disease status and physical conditions who should receive individualized therapeutic regimens, it is essential to identify different risk groups according to various biomarkers. Therefore, potential biomarkers are required and crucial for predicting the patient prognosis and designing therapeutic regimen and follow-up scheme.

The systemic inflammatory response (SIR), being associated with the outcome of a variety of tumor-related inflammation, is considered an important component of tumor progression [3]. Immune and inflammatory cells in peripheral blood, such as neutrophils, lymphocytes, platelets, and monocytes, play important roles in the tumor micro-environment and relate to invasion and metastasis of tumor cells [4]. Some indexes of the SIR-related cells, such as neutrophil to lymphocyte ratio (NLR), platelet-to-lymphocyte ratio (PLR), and monocyte to lymphocyte ratio (MLR), have been used to predict survival and recurrence of various cancers, including gastric cancer [5–8]. Among the indexes, PLR is considered a potential marker of endogenous residual anticancer pre-inflammatory and pre-coagulative response that arises in malignancies and is highly repeatable, cost-effective, and widely available [9, 10]. The application of PLR in the diagnosis and prognosis of gastric cancer was also reported in a variety of studies but with controversial results. For example, Kim et al. found that elevated PLR predicted poor overall survival (OS) and disease-free survival (DFS) in GC patients after surgery [11]. However, some other studies did not detect the significant prognostic value of PLR for GC patients [12, 13]. We conducted this meta-analysis to investigate the prognostic significance of pre-treatment PLR for OS and DFS, and the associations between PLR and clinicopathological features in GC patients.

## Materials and methods

### Literature search

**Fig. 1:**
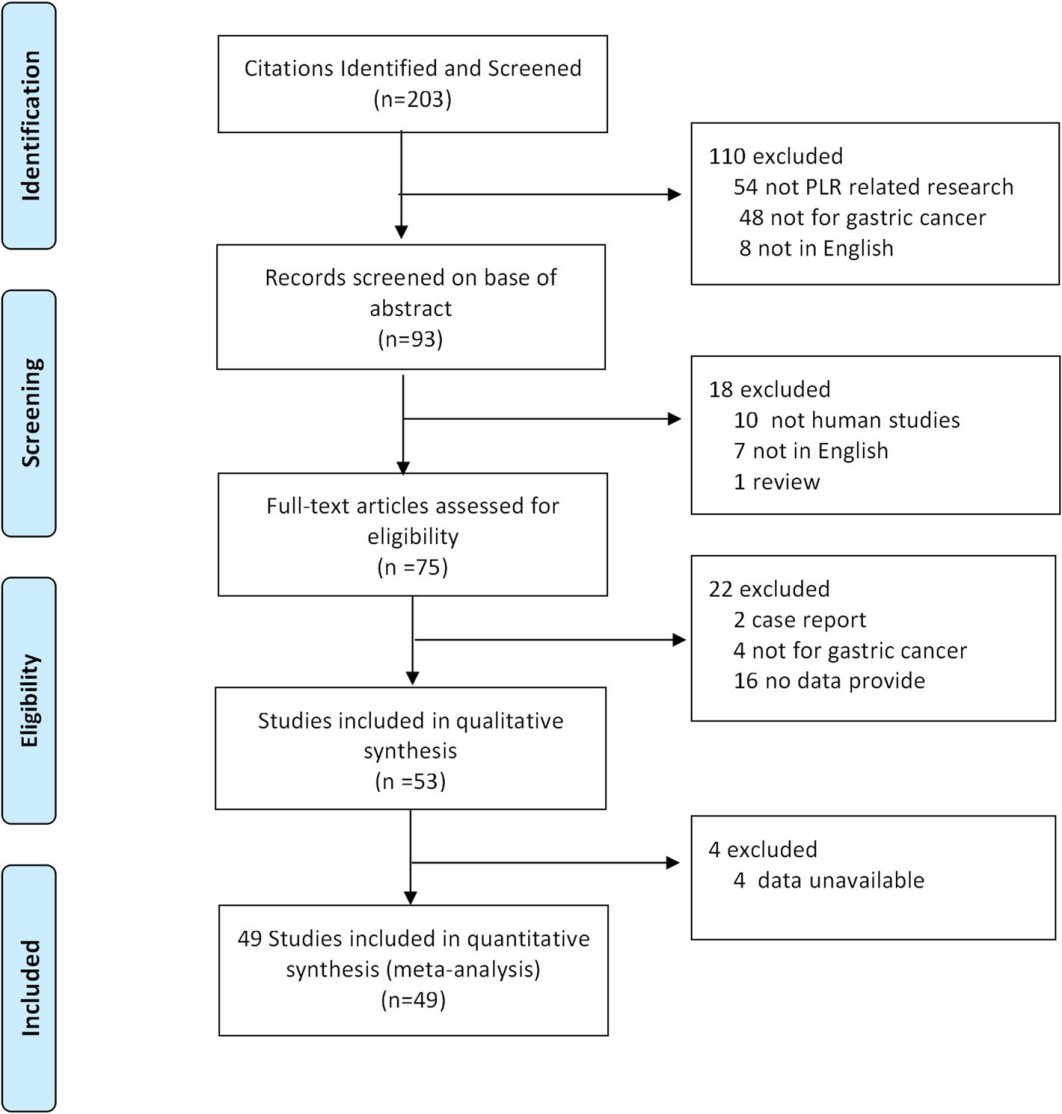
The flow diagram of publications selection

We performed a systematic literature search in PubMed, Embase, and the Cochrane Library. The search strategy terms are as follows: (PLR or “platelet lymphocyte ratio” or “platelet-to-lymphocyte ratio” or “platelet-lymphocyte ratio”) and (“gastric cancer” or “gastric adenocarcinoma” or “gastric carcinoma” or “GC” or “gastric neoplasm” or “stomach tumor” or “stomach neoplasm”). The last search was updated to April 8, 2020, and studies published in English were included. This study was conducted according to the Preferred Reporting Items for Systematic Reviews and Meta-Analyses (PRISMA) statement, and a flow chart of the systematic review is shown in Fig. 1. No ethical approval and patient consent are required in this study.

### Inclusion and exclusion criteria

The predetermined inclusion and exclusion criteria were applied for the including of the articles in this study: Inclusion criteria are as follows: (1) the diagnosis must be confirmed by pathological examination; (2) HR and 95% CI for the OS and (or) DFS, the number of patients with various clinicopathological features are available; (3) PLR is the result of pre-treatment. Exclusion criteria are as follows: (1) conference abstracts, reviews, letters to the editor, and other nonclinical literature are not applied; (2) articles with insufficient data to estimate are not included; and (3) the articles with non-human research or non-English language are not included.

### Data extraction and quality assessment

All studies were assessed independently by two authors according to the designed eligibility criteria. Any questions or disagreements were resolved by consulting another co-author. The extracted data included the following study information: first author, publication year, country, study design (retrospective or prospective), study period, treatment regimens, follow-up time, cut-off value of PLR, and the number of patients with various clinicopathological features, including tumor location, differentiation, size, depths of tumor invasion, lymph node metastasis, TNM stage, and HRs with 95% CIs of OS and DFS. The quality of each study was assessed according to the Newcastle-Ottawa Scale (NOS) by two authors [14] and a NOS score ≥ 6 were considered high-quality researches.

### Statistical analysis

The pooled HRs were calculated based on HRs and their 95% CIs from each study to estimate prognostic role of PLR in GC patients. HRs and 95% CIs for OS and DFS were obtained directly from each study if available or were calculated from the related data according to the methods published by Tierney et al. [15]. Cochran’s *Q* test and Higgins *I*-squared statistic were used to evaluate the heterogeneity of pooled results. A *p* value < 0.1 for the *Q* test or *Ι*^2^ > 50% indicate significant heterogeneity among studies, and the random-effects model (DerSimonian-Laird method) was performed to calculate the pooled HRs. Otherwise, the fixed-effects model (Mantel-Haenszel method) was applied [16]. Odds ratios (OR) and 95% CI were used to analyze the relationship between PLR and clinicopathological factors. Publication bias of the literature was evaluated by Begg’s funnel plot and Egger’s linear regression tests, and *p* > 0.05 indicated that there was no significant publication bias. Sensitivity analysis was performed by removing each single study in turn to validate the stability of the pooled results. All statistical analyses were performed using STATA software version 14.0 (STATA Corporation, College Station, TX, USA). Results with *p* < 0.05 were considered statistically significant, and all the results were two sided.

## Results

### Study characteristics

A total of 49 studies (51 cohorts) [7, 11–13, 17–61] with 28,929 GC patients were included in the final meta-analysis. As in Fan Feng’s study [37], the GC patients were included in a training set and a validation set independently; therefore, the two cohorts were extracted separately and named as Fan Feng(1) and Fan Feng(2). As in Aldemir’s study, GC patients were divided into the surgery group and chemotherapy group. So we named the two groups as Aldemir(1) and Aldemir(2) [60]. The selection process of the included studies according to the PRISMA guidelines was shown in Fig. 1. We summarized the characteristics of the studies in Table 1. Among them, 10 studies were from Europe and the USA and 41 studies from Asia. The patients from 27 studies received surgery treatment, the patients with an advanced stage from 8 studies received chemotherapy strategy, and the patients from other 6 studied received mixed treatment (including chemotherapy, surgery, radiotherapy, targeted therapy, and supportive care). The cut-off values of PLR among the studies varied from 10.1 to 350. Therefore, we selected PLR = 150 to divide the studies in subgroup analysis. All studies with NOS scores ≥ 6 were regarded as high-quality studies.

**Table 1:** Characteristics of included studies in meta-analysis

Author	Year	Country	Ethnicity	Treatment	Follow-up (month)	Cut-off	Study period	Patients (***n***)	Survival analysis	NOS score
Mehmet Aliustaoglu	2010	Turkey	Caucasian	Chemotherapy	NA	160	2004–2008	168	OS	7
Deshen Wang	2012	China	Asian	Surgery	39.9 (23.77–57.43)	150/300	2006–2009	324	OS/DFS	8
Suee Lee	2013	Korea	Asian	Chemotherapy	14.9 (1–47.9)	160	2007–2010	174	OS	7
Qing Wang	2014	China	Asian	Mixed	NA	160	2006–2014	439	OS	7
Dawei Yuan	2014	China	Asian	Surgery	NA	150	2009–2012	280	OS/DFS	7
Nan Jiang	2014	China	Asian	Surgery	42 (1–103)	184	2005–2007	377	OS	8
Lian Lian	2015	China	Asian	Surgery	60	208	2007–2010	162	OS/DFS	8
Fen Wang	2015	China	Asian	Chemotherapy	40	235	2010–2011	120	NA	6
KaiYu Sun	2015	China	Asian	Surgery	55.75 (0.8–186)	140	1998–2008	632	OS	8
Xuechao Liu	2015	China	Asian	Surgery	NA	180	2015–2010	455	OS	7
Meral Gunaldi	2015	Turkey	Caucasian	Mixed	11.5	160	NA	245	OS	6
M. Messager	2015	UK	Caucasian	Surgery	31.8 (4–131)	192	2001–2014	153	OS/DFS	8
Qiwen Deng	2015	China	Asian	Surgery	24 (3–60)	132	2007–2009	389	OS/DFS	8
Jun-Te Hsu	2015	China	Asian	Surgery	30	132	2005–2011	1030	OS	8
Eun Young Kim	2015	Korea	Asian	Surgery	NA	126	2000–2009	1986	OS/DFS	7
Aldemir(1)	2015	Turkey	Caucasian	Surgery	NA	170	2006–2013	53	OS	7
Aldemir(2)	2015	Turkey	Caucasian	Chemotherapy	NA	170	2006–2013	50	OS	7
Wenyang Pang	2016	China	Asian	Surgery	NA	155.67	2009–2011	492	NA	6
Xin Zhou	2016	China	Asian	Surgery	NA	167	2006–2008	451	OS	7
Jin Wang	2016	China	Asian	Chemotherapy	NA	201.6	2005–2013	273	OS	7
Neng Lou	2017	China	Asian	Surgery	NA	106	2006–2014	312	NA	6
Weipeng Gong	2017	China	Asian	Surgery	22 (8–67)	161	2007–2015	91	OS	8
Kenichi Inaoka	2017	Japan	Asian	Surgery	NA	71	1999–2016	312	NA	6
Masayuki Urabe	2017	Japan	Asian	Surgery	63.3	NA	1999–2014	1363	OS/DFS	8
Shubin Song	2017	China	Asian	Surgery	37 (3–108)	139.12	2007–2011	1990	OS	8
Fan Feng(1)	2017	China	Asian	Surgery	24.9 (1–75)	130.675	2008–2015	1621	OS	8
Fan Feng(2)	2017	China	Asian	Surgery	24.9 (1–75)	130.675	2008–2015	1622	OS	8
Kenji Mima Tsu	2017	Japan	Asian	Surgery	NA	200	2006–2016	33	OS	7
Kang Wang	2017	China	Asian	Surgery	45 (1–185)	120	1994–2005	444	OS	8
Harry E. Fuentes	2017	USA	Caucasian	Mixed	21.3 (9.5–42.6)	260	2010–2015	112	OS	7
Mikito Mori	2018	Japan	Asian	Surgery	37 (5–108)	149.4	2006–2017	100	NA	7
Hongtai Shi	2018	China	Asian	Surgery	36 (1–75)	135	2012–2014	688	OS	8
YuChen Pan	2018	China	Asian	Surgery	59.9	115	2008–2012	870	OS	8
Guangsheng Zhu	2018	China	Asian	Surgery	NA	117.78	2010–2016	248	OS	7
Hai-Jeon Yoon	2018	Japan	Asian	Surgery	34.5 (6.5–74.8)	10.1	2011–2016	134	OS/DFS	8
Yan Zhang	2018	China	Asian	Mixed	NA	172	2011–2014	182	OS/DFS	7
Ji lin	2018	China	Asian	Surgery	NA	116.85	2015–2016	670	OS	7
A. Ramos-Esquivel	2018	Costa Rica	Caucasian	Mixed	13.21 (0–84)	350	2009–2012	381	OS/DFS	7
Jiaxin Wen	2018	UK	Caucasian	Surgery	NA	150	2003–2015	668	OS	7
Angelica Petrillo	2018	Italy	Caucasian	Chemotherapy	29 (20.4–37.5)	157	2010–2017	151	OS	8
Hiroaki Saito	2018	Japan	Asian	Surgery	NA	173.3	2005–2013	453	OS	7
Cheng Tang	2018	China	Asian	Surgery	NA	130.7	2010–2016	104	OS	7
Li-xiang Zhang	2018	China	Asian	Surgery	NA	160	2010–2011	904	OS	7
Osama Abu-Shawer	2019	Jordan	Asian	Mixed	NA	150	NA	447	OS	7
Xinran Zhang	2019	China	Asian	Surgery	44.9 (1–188.9)	168.5	2000–2010	2752	OS	8
Cuixia Liu	2019	China	Asian	Surgery	NA	152.2	2009–2012	400	NA	6
Hua-Long Zheng	2019	China	Asian	Surgery	54 (35–67)	133.03	2009–2013	924	OS	8
Yuka Ohe	2020	Japan	Asian	Chemotherapy	NA	180	2005–2018	41	OS	7
Ibrahim Mungan	2020	Turkey	Caucasian	Surgery	NA	181.8	2015–2018	292	NA	6
Jian-Xian Lin	2020	China	Asian	Surgery	65.6 (1–117)	162.5	2009–2014	2257	OS	8
Guanghui Zhao	2020	China	Asian	Chemotherapy	11.6	143.39	2012–2016	110	OS	8

*NA* not available, *OS* overall survival, *DFS* disease-free survival, *NOS* Newcastle-Ottawa Scale

### PLR and prognosis of GC

**Fig. 2:**
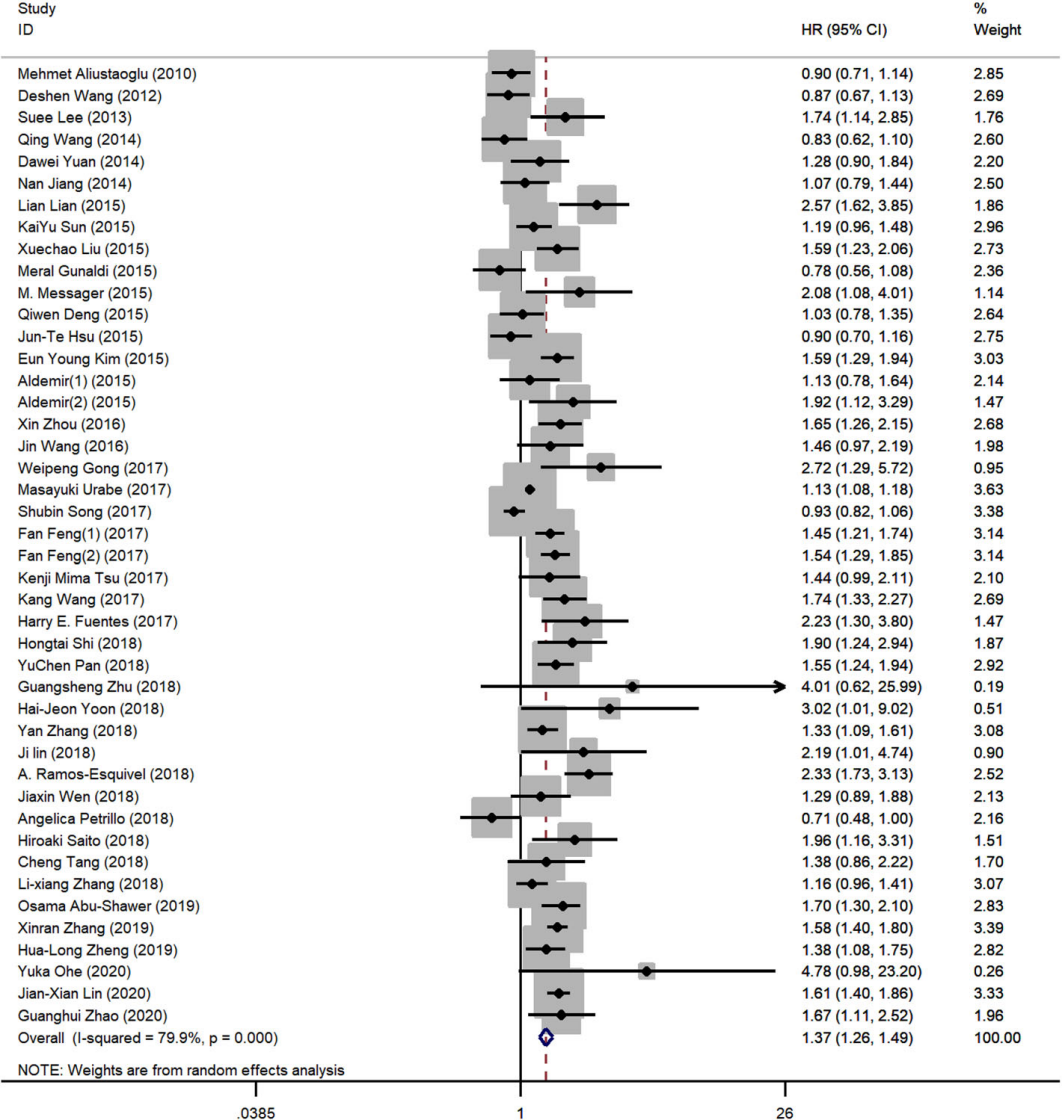
The forest plot between elevated PLR and OS in GC patients

PLR in 44 cohorts with 26,901 GC patients were evaluated for OS [7, 11–13, 17, 19–27, 29–34, 37, 38, 41–51, 53–61]. The main results of this meta-analysis are listed in Table 2. We found that elevated PLR was significantly associated with a worse outcome for OS (HR 1.37, 95% CI 1.26–1.49, *p* < 0.001), and significant heterogeneity was observed (*I*^2^ = 79.90%, *P*_h_ < 0.001, Table 2, Fig. 2).

**Table 2:** Main results of the meta-analysis

	Factors	No. of studies	No. of patients	Effects model	HR (95% CI)	***p***	Heterogeneity	
							***I***^**2**^	***P***_**h**_
**OS**	**Overall**	44	26901	Random	1.37 (1.26–1.49)	< 0.001	79.90%	< 0.001
	**Ethnicity**							
	Caucasian	9	1981	Random	1.31 (0.96–1.79)	0.092	84.10%	< 0.001
	Asian	35	24920	Random	1.39 (1.28–1.52)	< 0.001	79.20%	< 0.001
	**Treatment**							
	Chemotherapy	7	967	Random	1.34 (0.96–1.88)	0.084	76.10%	< 0.001
	Surgery	31	24128	Random	1.39 (1.26–1.52)	< 0.001	79.10%	< 0.001
	Mixed	6	1806	Random	1.38 (0.98–1.93)	0.062	88.20%	< 0.001
	**Cut-off**							
	≤ 150	20	15181	Random	1.36 (1.20–1.54)	< 0.001	75.00%	< 0.001
	> 150	23	10357	Random	1.42 (1.24–1.63)	< 0.001	78.50%	< 0.001
	**Sample size**							
	≤ 500	29	6924	Random	1.42 (1.24–1.64)	< 0.001	75.70%	< 0.001
	> 500	15	19977	Random	1.34 (1.20–1.50)	< 0.001	85.00%	< 0.001
**DFS**	**Overall**	10	5354	Random	1.52 (1.22–1.90)	< 0.001	88.60%	< 0.001

*HR* hazard ratio, *95% CI* 95% confidence interval, *P*_*h*_*p* values of *Q* test for heterogeneity test, *OS* overall survival, *DFS* disease-free survival

All patients were stratified by ethnicity, treatment, cut-off value of PLR, and sample size for subgroup analysis. The results showed that elevated PLR had more significantly prognostic value for OS in Asian populations (HR 1.39, 95% CI 1.28–1.52, *p* < 0.001; *I*^2^ = 79.20%, *P*_h_ < 0.001), but not in Caucasian populations. Furthermore, when different treatment methods were considered, elevated PLR significantly predicted shorter OS in patients receiving surgery treatment (HR 1.39, 95% CI 1.26–1.52, *p* < 0.001; *I*^2^ = 79.10%, *P*_h_ < 0.001) but have no prognostic efficiency for patients receiving chemotherapy or mixed treatment. Considering different cut-off values, both PLR with cut-off value > 150 (HR 1.42, 95% CI 1.24–1.63, *p* < 0.001; *I*^2^ = 78.50%, *P*_h_ < 0.001) and ≤ 150 (HR 1.36, 95% CI 1.20–1.54, *p* < 0.001; *I*^2^ = 75.00%, *P*_h_ < 0.001) predicted poor OS for GC. Of note, we found that PLR, as a negative prognostic marker, was significantly associated with the OS in GC patients both in sample size ≤ 500 groups (HR 1.42, 95% CI 1.24–1.64, *p* < 0.001; *I*^2^ = 75.70%, *P*_h_ < 0.001) and > 500 groups (HR 1.34, 95% CI 1.20–1.50, *p* < 0.001; *I*^2^ = 85.00%, *P*_h_ < 0.001; Table 2).

**Fig. 3:**
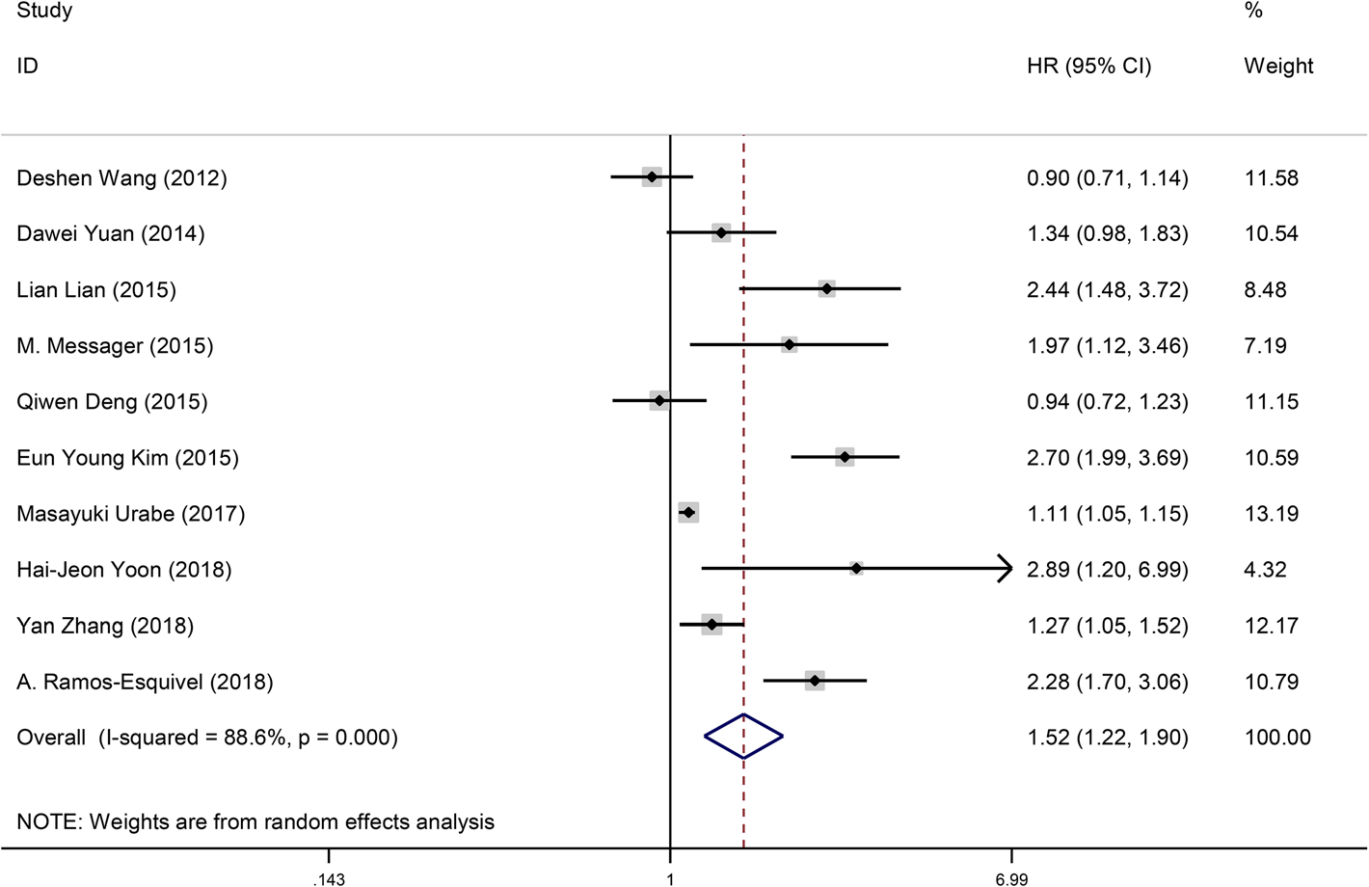
The forest plot between elevated PLR and DFS in GC patients

Ten studies with 5354 subjects explored the influence of PLR on DFS of GC patients [7, 11, 12, 20–22, 24, 26, 42, 44, 47]. The pooled data of our meta-analysis indicated that the PLR was associated with DFS (HR 1.52, 95% CI 1.22–1.90, *p* < 0.001, *I*^2^ = 88.6%, *P*_h_ < 0.001) (Table 2, Fig. 3).

### PLR and clinicopathological parameters of GC

To further explore the impact of PLR on the clinicopathological parameters in GC, we extracted the number of patients from parts of studies in PLR-high and PLR-low groups according to the TNM stage, tumor differentiation, depth of invasion, tumor size, tumor location, and lymph node metastasis. As shown in Table 3, in comparison to low PLR groups, the high PLR groups had a higher risk of lymph node metastasis (*n* = 15, OR = 1.17, 95% CI 1.02–1.33, *p* = 0.023), serosal invasion (T3+T4) (*n* = 13, OR = 1.34, 95% CI 1.10–1.64, *p* = 0.003), and increased advanced stage (III+IV) (*n* = 16, OR = 1.20, 95% CI 1.06–1.37, *p* = 0.004), whereas elevated PLR value was not shown to be associated with tumor size, tumor differentiation, and tumor location.

**Table 3:** Meta-analysis of the association between PLR and clinicopathological parameters of GC

Variable	No. of studies	No. of patients	Effects model	OR (95% CI)	***p***	Heterogeneity	
						***I***^**2**^	***P***_**h**_
**Tumor differentiation (moderate/high vs. poor)**	18	6721	Fixed	1.04 (0.98–1.11)	0.173	7.30%	0.367
**Tumor location (cardia vs. non-cardia)**	10	2905	Fixed	0.99 (0.87–1.12)	0.837	6.00%	0.386
**Tumor size (≤ 5 vs. > 5 cm)**	8	2596	Random	1.04 (0.88–1.23)	0.634	74.20%	< 0.001
**Lymph node metastasis (no vs. yes)**	15	6752	Random	1.17 (1.02–1.33)	0.023	71.90%	< 0.001
**Depth of invasion (T1–T2 vs. T3–T4 )**	13	6250	Random	1.34 (1.10–1.64)	0.003	86.20%	< 0.001
**TNM (Tis-II vs. III-IV)**	16	6834	Random	1.20 (1.06–1.37)	0.004	77.30%	< 0.001

*OR* odds ratio, *95% CI* 95% confidence interval, *P*_*h*_*p* values of *Q* test for heterogeneity test

### Sensitivity analysis

**Fig. 4:**
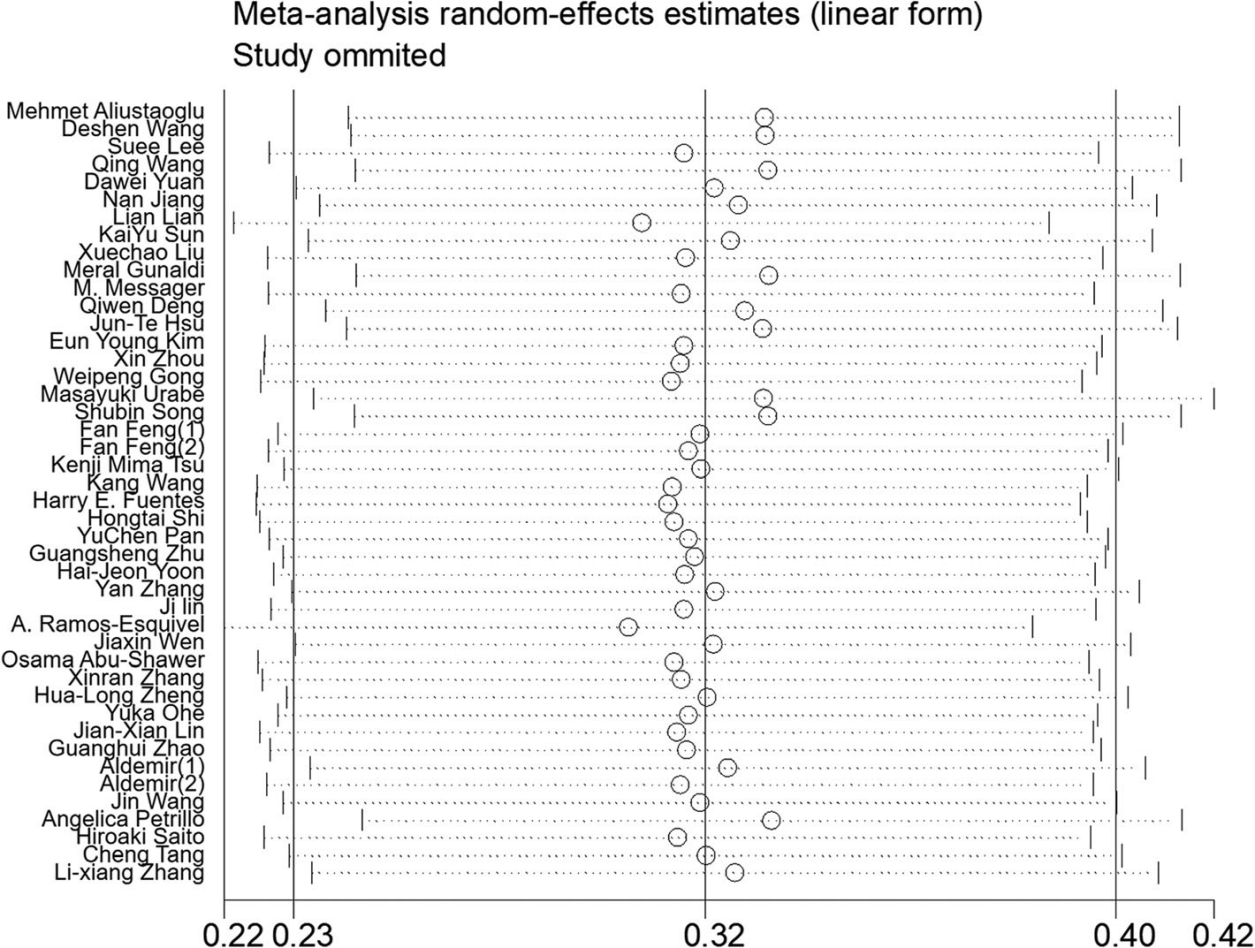
Sensitivity analysis of PLR for OS in GC patients

We performed sensitivity analysis for the OS by removing one single study at a time to check if individual study influenced the results. The corresponding pooled HRs are consistent, indicating stable and robust results in this meta-analysis (Fig. 4).

### Publication bias

**Fig. 5:**
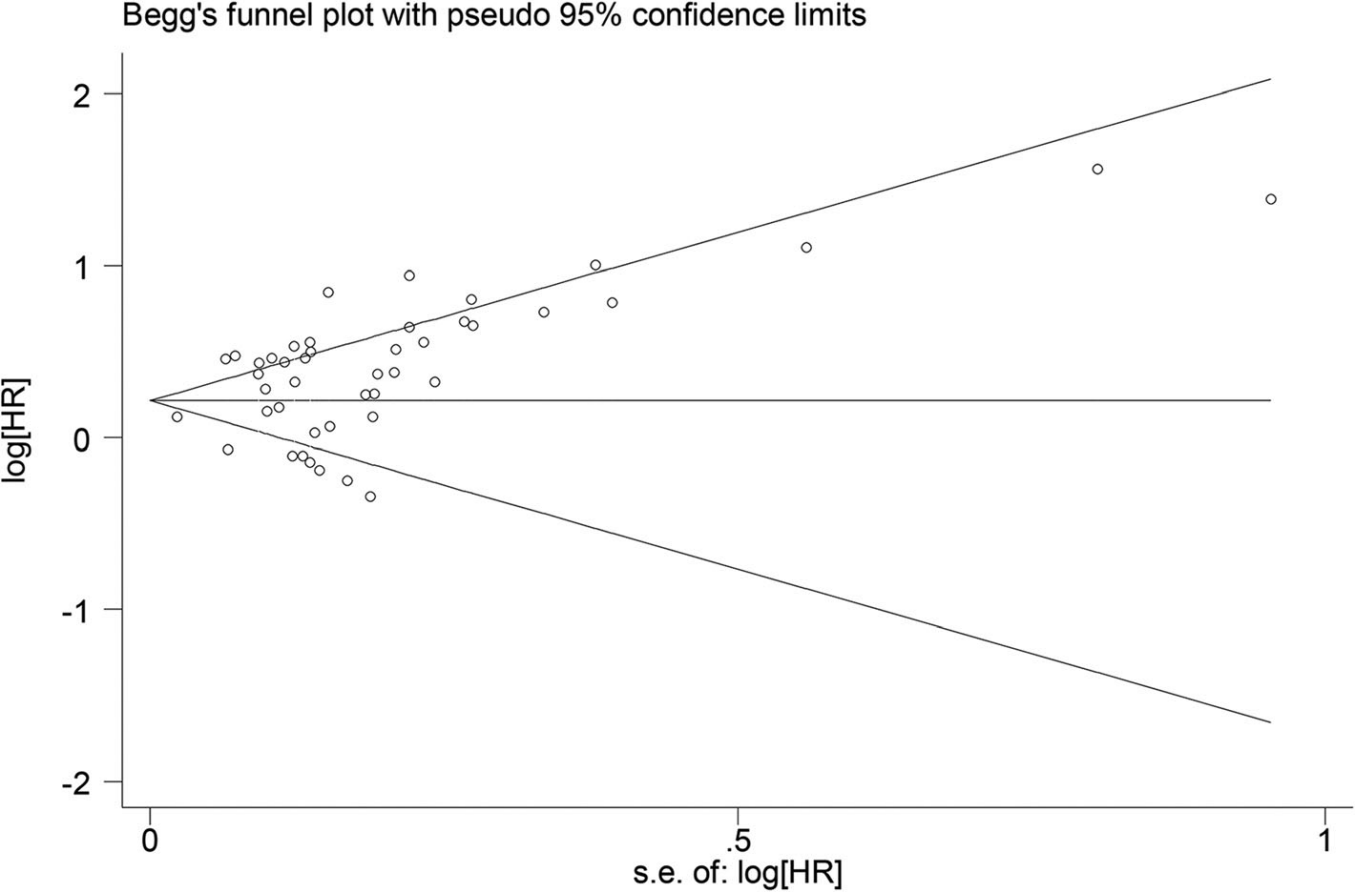
Begg’s funnel plot of publication bias test for OS in GC patients

Begg’s funnel plot and the Egger’s linear regression test were performed to assess publication bias. The figure of the Begg’s funnel plot showed obvious asymmetry (Fig. 5) and Egger’s tests (*p* = 0.004) indicated significant publication bias. However, our finding that elevated PLR is associated with lower OS did not change after the adjustment for publication bias using the trim and fill method [62].

## Discussion

The current meta-analysis was designed to investigate the prognostic value of elevated PLR for DFS and OS in GC patients. Pooled results demonstrated that elevated PLR was associated with poor OS and DFS. Moreover, elevated PLR was correlated with lymph node metastasis, serosal invasion, and advanced TNM stage with GC.

Despite the development of new surgical techniques and the use of chemotherapy and radiotherapy, gastric cancer still remains one of the main causes of cancer-related mortality and morbidity worldwide [63]. Because individual GC patients present with different conditions, including different degrees of invasion, differentiation, and TNM stages, the survival outcomes may vary. Therefore, identification of reliable prognostic factors, simple and low cost, to stratify patients into different risk groups, would contribute to the optimization of individualized treatment and follow-up. In recent years, the studies about the relationship between the inflammation and tumor have been developed. Inflammatory cells are critical factors in the tumor cell micro-environment and important for repair of tissue damage [64–66]. The inflammation is involved in lymphocytopenia, neutrophilia, thrombocytosis, and leukocytosis [67, 68]. The tumor-generated inflammatory reaction may contribute to tumor growth, progression, and metastasis through several mechanisms, including the upregulation of inflammatory mediators and cytokine, aberrant activation of immune regulatory cytokines, suppression of apoptosis, and DNA damage [65]. Recently, emerging evidence indicates that inflammatory reaction is an important factor for the initiation, progression, and prognosis of numerous cancers, including GC [69, 70]. *Helicobacter pylori* infection in GC is characterized by an inflammatory infiltrate, consisting mainly of neutrophils and T cells [71]. Moreover, circulating lymphocytes were reported that could reflect patient’s inflammatory status [72]. Thus, some inflammation-based parameters, such as lymphocyte count, systemic immune-inflammation index (SII), platelet-lymphocyte ratio (PLR), and neutrophil-lymphocyte ratio (NLR), have been used to predict survival and recurrence in cancer patients [44, 73–76].

The PLR, which combines platelet and lymphocyte counts, is a representative index of systemic inflammation and immune status [77, 78]. Accumulating evidence indicates the correlation of PLR with different stages of tumor development, chemotherapeutic response, and prognostic survival outcomes of GC patients [38, 42, 78]. The specific mechanisms involved are complex and remain unclear. One potential explanation is that a decreased PLR may reflect tumor disadvantage status, such as inflammatory status, immune disorders, malnutrition, and a tendency for micro-vessel thrombosis [39, 79]. Lymphocytes have an important role in cancer immune surveillance and preventing the development of malignancy [80]. A pro-inflammatory status leads to compromised cell-mediated immunity and impaired T-lymphocytic response via cytokines [81]. The decrease in CD4+ T-helper lymphocytes may result in a suboptimal lymphocyte-mediated immune response to tumor cells [82]. The T-lymphocytic cell-mediated malnutrition is a major cause of delayed wound healing [83, 84].

Platelet count is an additional index of a systemic inflammatory response and potential micro-vessel thrombosis, which could inhibit wound healing via the deterioration of blood circulation in tissues [11, 77, 85]. Otherwise, aggregated platelets can promote tumor growth via releasing pro-angiogenic mediators within the micro-vasculature of tumors [86]. Platelets also inhibit tumor cell extravasation by potentiating tumor cell-induced endothelial cell retraction, and enhance tumor cell adhesion and spreading across the extracellular matrix, which contribute to the promotion of tumor cell proliferation and metastasis [87]. Therefore, lymphocytopenia and thrombocytosis are considered negative prognostic markers in various cancers [88–91]. However, a decreased lymphocyte count or an increased platelet count alone may not reflect the host systemic inflammatory response and tumorigenesis process. Thus, the PLR, a biomarker combining platelet and lymphocyte counts, may better reflect the information of lymphocytopenia and thrombocytosis and predict the prognosis of GC patients. In addition, the value of PLR could be acquired from the routine laboratory tests, which provides clinical implications at a low cost.

Accumulated studies have assessed the association between PLR and the diagnosis and prognosis of gastric cancer. Some studies showed that elevated PLR predicted poor OS and DFS in GC patients after surgery [22, 24]. However, some other studies did not detect the significant prognostic value of PLR for GC patients [7, 47]. Lian et al. reported that low PLR levels correlated with better clinicopathological features, including decreased depth of invasion, less lymph node metastasis, and early tumor stage [44]. Recently, a meta-analysis containing 8 studies comprising 4513 patients was conducted and showed that PLR was not a reliable predictor for OS in patients with GC, while another meta-analysis including 13 studies with 6280 patients indicated that elevated PLR could be a significant prognostic biomarker for poor OS [92, 93]. Thus, the prognostic value of the PLR remains inconclusive in gastric cancer. So we conducted this updated meta-analysis to evaluate the prognostic role of the PLR in gastric cancer.

In the current study, including 49 studies (51 cohorts) with 28,929 GC patients, we not only investigated the prognostic value of PLR for OS and DFS, but also explored the associations between PLR and clinicopathological characteristics of GC. This analysis demonstrated that elevated PLR leads to a higher risk of lymph node metastasis, increased serosal invasion (T3+T4) risk, and advanced stage (III+IV) in patients with gastric cancer. Although the specific mechanism is still incompletely understood, our results are in accordance with other studies in various cancers, such as pancreatic ductal adenocarcinoma, hepatocellular carcinoma, and colorectal cancer [94–98].

Previous meta-analysis did not find significant association between PLR and OS or DFS in GC, maybe because of the limited studies included [92, 93]. Our meta-analysis including much more studies suggested that elevated PLR might have powerful prognostic efficiency for poor OS in GC and could predict shorter DFS in GC. Subgroup analyses for OS revealed the similar result in Asian populations, but not in Caucasian populations. Moreover, we also eliminated the effect of different treatment methods on the prognostic value of the PLR. Our results showed that elevated PLR significantly predicted shorter OS in patients receiving surgery treatment, but did not have prognostic efficiency for patients receiving chemotherapy or mixed treatment. Except for the reason of too few studies included, another possible major reason is that the patients in the chemotherapy or mixed groups have huge differences in medical conditions and disease status, resulting in the inability to obtain significant results. To evaluate the effect of different cut-off values on the prognostic value of PLR in GC patients, subgroup analyses showed that patients with elevated PLR suffered worse OS than those with low PLR, regardless of the different cut-off values. The same effects were indicated in the subgroup analyses by different sample size of patients. These results might strengthen the possibility that PLR could act as a reliable prognostic biomarker in GC.

There were some limitations requiring to be addressed in this meta-analysis. First, the inclusion criteria of this meta-analysis were constrained to studies published in the English language only. So publication bias cannot be excluded. Second, almost the studies included were all retrospective, which could be more susceptible to some biases. Fortunately, the asymmetry in the funnel plots showed no significant publication bias, thus maintaining the substantial consistency of the results. Third, the different cut-off values of PLR used in each study could contribute to the heterogeneity. Subgroup analysis was conducted based on the different PLR cut-off values, while the results were not substantially changed. Further well-designed studies, especially randomized controlled trials (RCTs), are needed to determine the most appropriate cut-off value of PLR to predict the complication risks and survival outcomes in patients with GC.

## Conclusions

In conclusion, elevated pre-treatment PLR is a prognostic factor for poor OS and DFS in GC patients. Furthermore, elevated PLR is correlated with a higher risk of serosal invasion, lymph node metastasis, and advanced TNM stage (III+IV) in gastric cancer. The present study suggests that the PLR could provide reliable information before treatment for patients with gastric cancer.

## Data Availability

All data for this study are publicly available and are ready for the public to download at no cost from the official websites of the PubMed, EMBASE, and the Cochrane Library. There is no need to have the formal permission to use data for this study. The sources and data robustness have been described in the “Materials and methods” section.

## Abbreviations

HR: Hazard ratio
OR: Odds ratio
95% CI: 95% Confidence interval
*P*_h_: *p* values of *Q* test for heterogeneity test
OS: Overall survival
DFS: Disease-free survival
PLR: Platelet-lymphocyte ratio
GC: Gastric cancer
SIR: Systemic inflammatory response
NLR: Neutrophil to lymphocyte ratio
MLR: Monocyte to lymphocyte ratio
NOS: Newcastle-Ottawa Scale
